# Supplementary material for: hictk: blazing fast toolkit to work with .hic and .cool files
Source: Bioinformatics. 2024 Jun 24;40(7):btae408. doi: 10.1093/bioinformatics/btae408 (PMC11216752; doi:10.1093/bioinformatics/btae408)
Supplement: btae408_Supplementary_Data [file btae408_supplementary_data.zip › 2023-hictk-paper-an-suppl-text-2.pdf]

hictk: blazing fast toolkit to work with .hic and .cool files

Supplementary Text 2

1. Patch applied to straw to return interactions sorted by genomic coordinates

```
diff --git a/C++/main.cpp b/C++/main.cpp
index 6a8061e..d192be5 100644
--- a/C++/main.cpp
+++ b/C++/main.cpp
@@ -21,6 +21,7 @@
  OUT OF OR IN CONNECTION WITH THE SOFTWARE OR THE USE OR OTHER DEALINGS IN
  THE SOFTWARE.
 */
+#include <algorithm>
 #include <iostream>
 #include <string>
 #include "straw.h"
@@ -48,6 +49,13 @@ int main(int argc, char *argv[])
     int32_t binsize = stoi(size);
     vector<contactRecord> records;
     records = straw(matrixType, norm, fname, chr1loc, chr2loc, unit,
binsize);
+    std::sort(records.begin(), records.end(),
+              [&](const contactRecord &r1, const contactRecord &r2) {
+                if (r1.binY == r2.binY) {
+                  return r1.binX < r2.binX;
+                }
+                return r1.binY < r2.binY;
+              });
     size_t length = records.size();
     for (int i = 0; i < length; i++) {
         printf("%d\t%d\t%.14g\n", records[i].binX, records[i].binY,
records[i].counts);
```

2. Patch applied to hicrep to use hictk instead of cooler

```
diff --git a/hicrep/__init__.py b/hicrep/__init__.py
index 80032bd..4f6e7b7 100644
--- a/hicrep/__init__.py
+++ b/hicrep/__init__.py
@@ -3,8 +3,9 @@ import numpy as np
 import math
```

```
 import sys
 import warnings
+import hictkpy
 from hicrep.utils import (
-    readMcool, cool2pixels, getSubCoo,
+    getSubCoo,
     trimDiags, meanFilterSparse, varVstran,
     resample
     )
@@ -20,16 +21,16 @@ def main(*args):
     np.random.seed(10)

     parser = argparse.ArgumentParser()
-    parser.add_argument("fmcool1", type=str,
-                        help="First cooler multiple-binsize contact files")
-    parser.add_argument("fmcool2", type=str,
-                        help="Second cooler multiple-binsize contact files")
+    parser.add_argument("file1", type=str,
+                        help="First .hic or .mcool contact file")
+    parser.add_argument("file2", type=str,
+                        help="Second .hic or .mcool contact file")
     parser.add_argument("fout", type=str,
                         help="Output results to this file. Output format
would be\
                         one column of scc scores for each chromosome")
-    parser.add_argument("--binSize", type=int, default=-1,
+    parser.add_argument("--binSize", type=int, default=0,
                         help="Use this to select the bin size from the input
mcool\
-                        file. Default to -1, meaning that the inputs are
treated as\
+                        file. Default to 0, meaning that the inputs are
treated as\
                         single-binsize .cool files")
     parser.add_argument("--h", type=int, required=True,
                         help="Smooth the input contact matrices using a 2d
mean\
@@ -54,7 +55,7 @@ def main(*args):
                         chromosomes whose names are provided. The output
SCC\
                         scores will be ordered as the input chromosome
names\
                         here")
-    parser.add_argument("--excludeChr", type=str, nargs='*', default=['M'],
+    parser.add_argument("--excludeChr", type=str, nargs='*',
default=['chrM', 'M'],
                         help="Exclude chromosomes from the SCC score
calculations.\
                         Mitochondrial chromosomes named \"M\" are excluded
by\
                         default. The output SCC scores will be ordered as
the\
@@ -63,7 +64,7 @@ def main(*args):
```

```
     args = parser.parse_args()

-    assert not (args.excludeChr != ['M'] and len(args.chrNames) > 0), f"""
+    assert not (args.excludeChr != ['chrM', 'M'] and len(args.chrNames) >
0), f"""
         Please use --chrNames OR --excludeChr arguments but not both.
         """

@@ -84,8 +85,6 @@ def main(*args):
             header += "# @rev " + p.sub("\n# @branch ",
                                         gitrev.stdout.read().strip()) + "\n"

-    fmcool1 = args.fmcool1
-    fmcool2 = args.fmcool2
     fout = args.fout
     binSize = args.binSize
     h = args.h
@@ -98,10 +97,10 @@ def main(*args):
         warnings.warn(f"""
             Duplicate excludeChr found. Please remove them in
--excludeChr""")

-    cool1, binSize1 = readMcool(fmcool1, binSize)
-    cool2, binSize2 = readMcool(fmcool2, binSize)
+    file1 = hictkpy.File(args.file1, binSize)
+    file2 = hictkpy.File(args.file2, binSize)

-    scc = hicrepSCC(cool1, cool2, h, dBPMax, bDownSample,
+    scc = hicrepSCC(file1, file2, h, dBPMax, bDownSample,
                     chrNames if len(chrNames) > 0 else None,
                     excludeChr if len(excludeChr) > 0 else None)

diff --git a/hicrep/hicrep.py b/hicrep/hicrep.py
index 69052b0..064f18b 100644
--- a/hicrep/hicrep.py
+++ b/hicrep/hicrep.py
@@ -20,11 +20,11 @@ import scipy.sparse as sp
 import math
 import sys
 import warnings
-import cooler
+import hictkpy
 from hicrep.utils import (
-    readMcool, cool2pixels, getSubCoo,
+    getSubCoo,
     trimDiags, meanFilterSparse, varVstran,
-    resample, upperDiagCsr, coolerInfo
+    resample, upperDiagCsr, fileInfo
     )

 @deprecated("Use sccByDiag instead")
```

```
@@ -91,14 +91,14 @@ def sccByDiag(m1: sp.coo_matrix, m2: sp.coo_matrix,
nDiags: int):
     return rhoNan2Zero @ wsNan2Zero / wsNan2Zero.sum()


-def hicrepSCC(cool1: cooler.api.Cooler, cool2: cooler.api.Cooler,
+def hicrepSCC(f1: hictkpy.File, f2: hictkpy.File,
               h: int, dBPMax: int, bDownSample: bool,
               chrNames: list = None, excludeChr: set = None):
     """Compute hicrep score between two input Cooler contact matrices

     Args:
-        cool1: `cooler.api.Cooler` Input Cooler contact matrix 1
-        cool2: `cooler.api.Cooler` Input Cooler contact matrix 2
+        f1: `cooler.api.Cooler` Input Cooler contact matrix 1
+        f2: `cooler.api.Cooler` Input Cooler contact matrix 2
         h: `int` Half-size of the mean filter used to smooth the
         input matrics
         dBPMax `int` Only include contacts that are at most this genomic
@@ -114,19 +114,17 @@ def hicrepSCC(cool1: cooler.api.Cooler, cool2:
cooler.api.Cooler,
     Returns:
         `float` scc scores for each chromosome
     """
-    binSize1 = cool1.binsize
-    binSize2 = cool2.binsize
-    assert binSize1 == binSize2,\
+    assert f1.bin_size() == f2.bin_size(),\
         f"Input cool files have different bin sizes"
-    assert coolerInfo(cool1, 'nbins') == coolerInfo(cool2, 'nbins'),\
+    assert f1.nbins() == f2.nbins(),\
         f"Input cool files have different number of bins"
-    assert coolerInfo(cool1, 'nchroms') == coolerInfo(cool2, 'nchroms'),\
+    assert f1.nchroms() == f2.nchroms(),\
         f"Input cool files have different number of chromosomes"
-    assert (cool1.chroms()[:] == cool2.chroms()[:]).all()[0],\
+    assert f1.chromosomes() == f2.chromosomes(),\
         f"Input file have different chromosome names"
-    binSize = binSize1
-    bins1 = cool1.bins()
-    bins2 = cool2.bins()
+    binSize = f1.bin_size()
+    bins1 = f1.bins()
+    bins2 = f2.bins()
     if binSize is None:
         # sometimes bin size can be None, e.g., input cool file has
         # non-uniform size bins.
@@ -142,20 +140,23 @@ def hicrepSCC(cool1: cooler.api.Cooler, cool2:
cooler.api.Cooler,
                        f"to determine maximal diagonal index to include",
RuntimeWarning)
     if dBPMax == -1:
         # this is the exclusive upper bound
```

```
-         dMax = coolerInfo(cool1, 'nbins')
+         dMax = f1.bins().shape[0]
     else:
         dMax = dBPMax // binSize + 1
     assert dMax > 1, f"Input dBPmax is smaller than binSize"
-    p1 = cool2pixels(cool1)
-    p2 = cool2pixels(cool2)
     # get the total number of contacts as normalizing constant
-    n1 = coolerInfo(cool1, 'sum')
-    n2 = coolerInfo(cool2, 'sum')
+    n1 = fileInfo(f1, 'sum')
+    n2 = fileInfo(f2, 'sum')
     # Use dict here so that the chrNames don't duplicate
     if chrNames is None:
-        chrNamesDict = dict.fromkeys(cool1.chroms()[:]['name'].tolist())
+        chrNamesDict = f1.chromosomes()
     else:
         chrNamesDict = dict.fromkeys(chrNames)
+
+    if "ALL" in chrNamesDict:
+        chrNamesDict.pop("ALL")
+    if "All" in chrNamesDict:
+        chrNamesDict.pop("All")
     # It's important to preserve the order of the input chrNames so that the
     # user knows the order of the output SCC scores so we bail when
encounter
     # duplicate names rather than implicit prunning the names.
@@ -169,11 +170,11 @@ def hicrepSCC(cool1: cooler.api.Cooler, cool2:
cooler.api.Cooler,
     scc = np.full(len(chrNames), -2.0)
     for iChr, chrName in enumerate(chrNames):
         # normalize by total number of contacts
-        mS1 = getSubCoo(p1, bins1, chrName)
+        mS1 = getSubCoo(f1, chrName)
         assert mS1.size > 0, "Contact matrix 1 of chromosome %s is empty" %
(chrName)
         assert mS1.shape[0] == mS1.shape[1],\
             "Contact matrix 1 of chromosome %s is not square" % (chrName)
-        mS2 = getSubCoo(p2, bins2, chrName)
+        mS2 = getSubCoo(f2, chrName)
         assert mS2.size > 0, "Contact matrix 2 of chromosome %s is empty" %
(chrName)
         assert mS2.shape[0] == mS2.shape[1],\
             "Contact matrix 2 of chromosome %s is not square" % (chrName)
diff --git a/hicrep/utils.py b/hicrep/utils.py
index a81a61a..10a3ebc 100644
--- a/hicrep/utils.py
+++ b/hicrep/utils.py
@@ -11,66 +11,13 @@ from typing import Union
 from contextlib import suppress
 import numpy as np
 import pandas as pd
-import cooler
```

```
-import h5py
+import hictkpy
 import math
 import scipy.sparse as sp

-def readMcool(fmcool: str, binSize: int):
-    """Read from a mcool or cool file and return the Cooler object

-    Args:
-        fmcool: Input file name
-        binSize: Bin size to select from the mcool file. If this value
-        is <= 0, the input will be treated as a cool file instead
-
-    Returns:
-        cooler.api.Cooler object
-    """
-    mcool = h5py.File(fmcool, 'r')
-    if binSize > 0:
-        return cooler.Cooler(mcool['resolutions'][str(binSize)]), binSize
-    else:
-        cool = cooler.Cooler(mcool)
-        return cool, cool.binsize
-
-
-def cool2pixels(cool: cooler.api.Cooler):
-    """Return the contact matrix in "pixels" format
-
-    Args:
-        cool: Input cooler object
-
-    Returns:
-        cooler.core.RangeSelector2D object
-    """
-    return cool.matrix(as_pixels=True, balance=False, sparse=True)
-
-
-def pixels2Coo(df: pd.DataFrame, bins: pd.DataFrame):
-    """Convert Cooler's contact matrix in "pixels" DataFrame to
-    scipy coo_matrix. The "pixels" format is a 3-column DataFrame:
-    'bin1_id', 'bin2_id', 'counts' for each unique contact
-
-    Args:
-        df: Input DataFrame
-        bins: Cooler bins for the contacts
-
-    Returns:
-        coo_matrix of the input
-    """
-    binOffset = bins.index[0]
-    nBins = bins.shape[0]
-    df['bin1_id'] -= binOffset
-    df['bin2_id'] -= binOffset
-    return sp.coo_matrix((df['count'].to_numpy(),
```

```
-                            (df['bin1_id'].to_numpy(),
df['bin2_id'].to_numpy())),
-                           shape=(nBins, nBins))
-
-
-def getSubCoo(pixels: cooler.core.RangeSelector2D, bins:
cooler.core.RangeSelector1D,
-               regionStr: str):
-    """Fetch a region from Cooler a contact matrix and return it as a
+def getSubCoo(f: hictkpy.File, regionStr: str):
+    """Fetch a region from a .hic or Cooler contact matrix and return it as
a
     coo_matrix

     Args:
@@ -81,12 +28,11 @@ def getSubCoo(pixels: cooler.core.RangeSelector2D, bins:
cooler.core.RangeSelect
     Returns:
         coo_matrix contact matrix corresponding to the input region
     """
-    mSub = pixels.fetch(regionStr)
+    mSub = f.fetch(regionStr).to_coo()
     # Assume Cooler always use upper triangle
-    assert (mSub['bin1_id'] <= mSub['bin2_id']).all(),\
+    assert (mSub.row <= mSub.col).all(),\
         f"Contact matrix of region {regionStr} has lower-triangle entries"
-    binsSub = bins.fetch(regionStr)
-    return pixels2Coo(mSub, binsSub)
+    return mSub


 def trimDiags(a: sp.coo_matrix, iDiagMax: int, bKeepMain: bool):
@@ -211,7 +157,7 @@ def resample(m: sp.coo_matrix, size: int):
     return ans


-def coolerInfo(cool: cooler.api.Cooler, k: str):
+def fileInfo(f: hictkpy.File, k: str):
     """Retrieve metadata from Cooler file

     The required metadata fields are documented in:
@@ -227,15 +173,14 @@ def coolerInfo(cool: cooler.api.Cooler, k: str):
         k (str): Key of the metadata field
     Returns: Requested metadata
     """
-    if k in cool.info:
-        return cool.info[k]
-    elif k == 'sum':
-        return cool.pixels()['count'][:].sum()
+    if k == 'sum':
+        attrs = f.attributes()
+        if 'sum' in attrs:
+            return attrs['sum']
```

```diff
+           return f.fetch().sum()
     elif k == 'nbins':
-           return cool.bins().shape[0]
-     elif k == 'nnz':
-           return cool.pixels().shape[0]
+           return f.nbins()
     elif k == 'nchroms':
-           return cool.chroms().shape[0]
+           return f.nchroms()
     else:
           raise KeyError(f'Unable to retrieve metadata field \'{k}\'')
diff --git a/setup.py b/setup.py
index 8f74363..f2367cd 100644
--- a/setup.py
+++ b/setup.py
@@ -38,7 +38,7 @@ setuptools.setup(
           "Deprecated",
           "numpy>=1.17.0",
           "scipy",
-          "cooler",
+          "hictkpy",
           "pandas",
           "h5py",
     ],
```
